# Correction: Effects of transboundary PM_2.5_ transported from China on the regional PM_2.5_ concentrations in South Korea: A spatial panel-data analysis

**DOI:** 10.1371/journal.pone.0315517

**Published:** 2024-12-05

**Authors:** Myung-Jin Jun, Yu Gu

The two authors, Myung-Jin Jun and Yu Gu, should be noted as contributing equally to this work.
