# Supplementary material for: Effects of transboundary PM2.5 transported from China on the regional PM2.5 concentrations in South Korea: A spatial panel-data analysis
Source: PLoS One. 2023 Apr 13;18(4):e0281988. doi: 10.1371/journal.pone.0281988 (PMC10101448; doi:10.1371/journal.pone.0281988)
Supplement: Attachment — Submitted filename: Report PONE-D-21-36719.pdf [file pone.0281988.s001.pdf]

Referee Report on: "Effects of transboundary PM2.5 transported from Chinese cities on the regional PM2.5 concentrations in South Korea: A spatial panel-data analysis"

1. Summary

This paper studies empirically how the long-range transboundary transport (LRTT) of PM2.5 from Chinese cities affects PM2.5 concentrations in South Korea. First, the author presents the spatial patterns of PM2.5 transport from China to South Korea using the hybrid single-particle Lagrangian integrated trajectory (HYSPLIT) model, which helps to identify the source city and arrival time of air mass in South Korean. Then, the spatial panel data regression is employed to estimate the marginal effects of LRTT from four different Chinese cities separately. The main finding is that the transported PM2.5 from three China cities has a major contribution to the regional PM2.5 concentration in South Korea. This finding is consistent with previous studies.

2. Evaluation

The effect of transboundary sources on regional PM2.5 concentrations is of great interest in literature. The existing models in literature estimate transboundary air pollution with data from satellite measurements or chemistry models. The contribution of this paper is to provide a direct statistical evidence of the effect of transboundary sources on regional PM concentrations with a more precise dataset from ground observations. This paper combines two datasets from the source cities in China and South Korea together. Overall, I think this paper makes a nice contribution to the literature. But the paper still needs improvement in some aspects for publication.

3. Main Comments

My main comments are as follows.

- In spatial panel regression, LRTT variables from those Chinese cities are not clear to me. According to the author, LRTT variables are determined by the observed PM2.5 concentration from the source region at the departure time. The author does not discuss the details of the travel time of the PM2.5 in HYSPLIT model. The only information readers can find is the travel time depends on the air mass travel speed and direction, and the maximum of the travel time is 5 days in HYSPLIT model. That means, at any time t, I do not know for which time the PM2.5 concentration from the source region is observed.

- One of my major concerns is about the endogeneity. It seems that the PM2.5 from Beijing will also affect the PM2.5 concentration in Shenyang by forward trajectories in the Figure 3. If this effect of transboundary sources among source cities is ignored, then the estimation of marginal effects of LRTT from a specific source city on local PM2.5 concentration is not valid.

- It would be more helpful if the author can discuss the insignificance of estimated coefficient of LRTT variables for Shenyang. Compared with other three regressions, regional meteorological factors plays an important role in the last regression. The insignificance of coefficient can be caused by other factor. For example, does the PM2.5 from Shenyang has a longer travel time than other cities?

- The author needs to reorganize the structure of the paper. The analysis of identification of source cities by HYSPLIT model should be presented before the econometric model setup. Then the summary statistics of the dependent variables and predictors follows. Figure 2 depicts the patterns of PM2.5 in source cities and South Korea. It can be placed before the test of random effects vs. fixed effects.

4. Minor Comments

- In Line 206, k is not defined. Be specific about the dimension of explanatory variables.
- In Table 3, some variables' name are confusing, e.g., $\mathbf{P}$ and Λ.
