# Supplementary material for: Effects of transboundary PM2.5 transported from China on the regional PM2.5 concentrations in South Korea: A spatial panel-data analysis
Source: PLoS One. 2023 Apr 13;18(4):e0281988. doi: 10.1371/journal.pone.0281988 (PMC10101448; doi:10.1371/journal.pone.0281988)
Supplement: Attachment — Submitted filename: Report2-PONE-D-21-36719.pdf [file pone.0281988.s004.pdf]

Second Round Referee Report on: "Effects of transboundary PM2.5 transported from Chinese cities on the regional PM2.5 concentrations in South Korea: A spatial panel-data analysis"

1. Evaluation

After the first round of review, the authors significantly improved the paper. This paper uses spatial panel-data models to statistically measure the effect of long-range transboundary transport (LRTT) of PM2.5 from China on PM2.5 levels in Korea. The backward trajectory pathways identify the source of the LRTT variable from China. Overall, I think this paper can be published conditional on clarifying several points.

2. Main Comments

- In line 307, it would be better to clarify the main predictor variables "LRTT of PM2.5 from China". For some N, at time t, is LRTT of PM2.5 from China defined as the average PM2.5 LRTT of China cities on HYSPLT back-trajectory five days before? I do not find a formal definition of this predictor variable, though the author discusses LRTT of PM2.5 from China on page 9.
- The author should explain more about Figure 2. One question is that the closest China city is 250km far away from the receptor Korean cities, by Figure 1. How can the author get the weekly average PM2.5 concentrations observed in Chinese cities in the pathways at a distance of 250km?

3. Minor Comments

- In line 243, I guess that the author is discussing Figure 5 instead of Figure 4.
- In line 167, I cannot see Figure 2 indicates that as the westerly wind approaches 168 Korea, it passes through these large cities,
