# Supplementary material for: Effects of transboundary PM2.5 transported from China on the regional PM2.5 concentrations in South Korea: A spatial panel-data analysis
Source: PLoS One. 2023 Apr 13;18(4):e0281988. doi: 10.1371/journal.pone.0281988 (PMC10101448; doi:10.1371/journal.pone.0281988)
Supplement: Attachment — Submitted filename: Report3-PONE-D-21-36719.pdf [file pone.0281988.s006.pdf]

Third Round Referee Report on: "Effects of transboundary PM2.5 transported from Chinese cities on the regional PM2.5 concentrations in South Korea: A spatial panel-data analysis"

1. Evaluation

After the second round of review, the authors significantly improved the paper and addressed issues from reviewers. This paper uses spatial panel-data models to statistically measure the effect of long-range transboundary transport (LRTT) of PM2.5 from China on PM2.5 levels in Korea. Overall, I think this paper can be published after minor revision.

2. Main Comments

(1) In the section "Synoptic Meteorological Conditions During the High-PM2.5 Episode", it is reasonable to believe that westerly winds and air pressure contributes to long-range transboundary transport (LRTT). I recommend author to illustrate why we need to look at the temperature and wind speed. It would be great to explain why relatively high temperature and stagnant air flow in Korea are considered to be the cause of high PM2.5 LRTT from China and high PM2.5 concentrations in Korea. To my knowledge, wind speed only changes the total time of LRTT transportation but not the amount of LRTT.

(2) In the description of the SEM model (Line 362-364), if the lag variable is used, it is better to have the notation like $X_{N(t-1)}$. One concern to me is that since the typical travel time of LRTT is five days, it is more reasonable to have an independent variables of LRTT with different lag periods.

(3) I recommend the author to mathematically formulate the computation of the average effects of LRTT from China on the local PM2.5 concentrations (maybe in a footnote). The first step how to get the marginal effect from the estimated coefficients.
