# Supplementary material for: Effects of transboundary PM2.5 transported from China on the regional PM2.5 concentrations in South Korea: A spatial panel-data analysis
Source: PLoS One. 2023 Apr 13;18(4):e0281988. doi: 10.1371/journal.pone.0281988 (PMC10101448; doi:10.1371/journal.pone.0281988)
Supplement: Attachment — Submitted filename: Comments_Reply_0829_22.docx [file pone.0281988.s003.docx]

Dear Reviewer,

Thank you for your insightful comments and feedback on the manuscript, which have enriched the manuscript and produced a better and more balanced account of the research. The following revisions were made to fulfill the reviewers’ requirements. I believe they have added to the value and quality of the manuscript.

1. The revised version uses a back-trajectory model that identifies air flows from China to nine receptor cities in Korea, while the original version of the manuscript used the forward-trajectory model starting from 12 source cities in China to analyze the effect of transboundary PM_2.5_ transported from these cities on the PM_2.5_ concentrations in South Korea. As we changed from forward-trajectory to back-trajectory, the focus of our analysis also changed from LRTT effects from source cities in China to effects on receptor cities in Korea.
2. Along with the back-trajectory model and the PM_2.5_ concentration observed in the monitoring stations in China and Korea, we collected satellite images retrieved from the Moderate Resolution Imaging Spectroradiometer (MODIS) Aerosol Optical Depth (AOD) and the European Copernicus Atmosphere Monitoring Service (CAMS) to identify causal links of a high-PM_2.5_ episode in Korea with air pollutants originated from China.
3. We analyzed the meteorological conditions favorable for maintenance of the high-PM_2.5_ episode in Korea, such as air pressure, wind direction and speed, and ambient temperature in both China and Korea.
4. We have provided a detailed explanation on determining PM_2.5_ levels originating from China. We found that the levels of PM_2.5_ observed in China on the back-trajectory pathways starts to increase from around 1,200 km and worsens as it passes 1,000 km away from the receptor cities. Therefore, in this study we use the average PM_2.5_ concentration observed in Chinese cities in the pathways identified by the HYSPLIT backward trajectories within 1,000 km from the receptor cities as the LRTT-related variable.
5. We have described in detail the process for finding the optimal spatial panel data model. As mentioned in the main text, we compared the Akaike Information Criteria (AIC) for the spatial error model (SEM) and the spatial autoregressive model (SAR) by testing seven different spatial weight matrices for the SEM and SAR models: queen contiguity, three distance-based (2, 4, and 8 k-nearest-neighbors (KNN)) and three kernel functions (uniform, triangular, and gaussian). We finally selected the SEM models with the 8-KNN spatial weight matrix as the most appropriate models, as these have the lowest AIC.
6. The introduction, conclusion, and policy implications have been rewritten according to the revised analysis results to emphasize the significance and distinction of the current study.
7. We have reorganized the structure of the paper by grouping the analysis into two parts: descriptive and visual analysis, and spatial panel-data analysis.

Reviewer #1:

| Comments | Reply and Revisions |
| --- | --- |
| In spatial panel regression, LRTT variables from those Chinese cities are not clear to me. According to the author, LRTT variables are determined by the observed PM_2.5_ concentration from the source region at the departure time. The author does not discuss the details of the travel time of the PM_2.5_ in HYSPLIT model. The only information readers can find is the travel time depends on the air mass travel speed and direction, and the maximum of the travel time is five days in HYSPLIT model. That means, at any time *t*, I do not know for which time the PM_2.5_ concentration from the source region is observed. | Thank you for your helpful comments. We have provided a detailed explanation on determining PM_2.5_ levels originating from China. We found that the levels of PM_2.5_ observed in Chinese cities on the back-trajectory pathways start to increase from around 1,200 km and worsen as they pass 1,000 km away from the receptor cities. Therefore, this study uses the average PM_2.5_ concentration observed in China in the pathways identified by the HYSPLIT backward trajectories within 1,000 km from the receptor cities as the LRTT-related variable. |
| One of my major concerns is about the endogeneity. It seems that the PM_2.5_ from Beijing will also affect the PM_2.5_ concentration in Shenyang by forward trajectories in the Figure 3. If this effect of transboundary sources among source cities is ignored, then the estimation of marginal effects of LRTT from a specific source city on local PM_2.5_ concentration is not valid. | As the focus of the analysis of this study changes from the source cities in China to the LRTT effects on receptor cities in Korea, we think there would be no endogeneity problems. |
| It would be more helpful if the author can discuss the insignificance of estimated coefficient of LRTT variables for Shenyang. Compared with other three regressions, regional meteorological factors plays an important role in the last regression. The insignificance of coefficient can be caused by other factors. For example, does the PM_2.5_ from Shenyang have a longer travel time than other cities? | As we changed from forward-trajectory to back-trajectory, the focus of the analysis also changed from LRTT effects from source cities in China to effects on receptor cities in Korea. |
| The author needs to reorganize the structure of the paper. The analysis of identification of source cities by HYSPLIT model should be presented before the econometric model setup. Then the summary statistics of the dependent variables and predictors follows. Figure 2 depicts the patterns of PM_2.5_ in source cities and South Korea. It can be placed before the test of random effects vs. fixed effects. | We have reorganized the structure of the paper following the reviewer’s helpful suggestions. We grouped the analysis parts into two: descriptive and visual analysis and spatial panel-data analysis. The descriptive and visual analysis includes: 1) analysis of a causal link between high-PM_2.5_ concentration in Korea and the LRTT-related PM_2.5_ from China, 2) synoptic meteorological conditions, 3) observed PM_2.5_ concentrations in China and Korea and back-trajectory pathways, and 4) aerosol optical depth (AOD) and CAMS PM_2.5_ concentration with wind direction. |
| In Line 206, *k* is not defined. Be specific about the dimension of explanatory variables. | Following your helpful suggestion, we added specific notations on N, t, and k. |
| In Table 3, some variables’ names are confusing, e.g., **P** and _. | A revision has been made accordingly. |

Reviewer #2:

| Comments | Reply and Revisions |
| --- | --- |
| 1. For such spatial dynamic analysis, the modeler shall include all endogenous variables in the model. The author only considered several cities from China and South Korea. It might be the case that cities from Japan and North Korea also contribute to this transboundary dynamic. Simple omission of these possible endogenous variables may lead to severe bias. | We thank you for your insightful comments. This study identified air flows using the back-trajectory model to find the source region of LRTT and found a causal link between the high concentration of PM_2.5_ in Korea and the LRTT from China. This finding was also confirmed by MODIS-AOD and CAMS satellite image data. This study also analyzed the meteorological conditions favorable for maintenance of the high-PM_2.5_ episode in Korea, such as air pressure, wind direction and speed, and ambient temperature. We included these meteorological variables into the spatial panel-data model along with the LRTT variable and secondary particles such as sulfur dioxide (SO_2_) and nitrogen oxides (NO_x_). |
| 2. The author has a wrong interpretation of the coefficient rho and lambda. The significance of these coefficients only suggests there exists spatial dynamic among these cities. It does not deliver the information of transboundary direction. | As you argue, the coefficient rho reflects the existence of spatially correlated errors, regardless the transboundary direction. In this study, the LRTT-related PM_2.5_ level is determined by the average PM_2.5_ concentration observed in China in the pathways identified by the back-trajectories within 1,000 km from the receptor cities, which includes the information of transboundary direction. |
| 3. The Hausman test to distinguish between fixed effect and random effect is largely applicable to cross sectional setting. I don’t see how the author applied it to spatial panel setting. | We refer to Belotti et al. (2017), which gives instruction on the Hausman test for the spatial panel-data model using *Stata,* even though it is allowed only for static models.  Reference:  Belotti et al. (2017) Spatial panel-data models using Stata, *The Stata Journal*, 17(1), pp. 139–180 |
| 4. It is confusing to read the summary statistics in Table 1, where variables appear in both rows and columns. | As we changed the focus of the analysis from LRTT effects from source cities in China to effects on receptor cities in Korea, Table 1 has been changed accordingly. |
| 5. The conjecture of transboundary effects from China to Korea based on Figure 2 is far from serious. | As we changed from forward-trajectory to back-trajectory, we revised Figure 2. In the current version, Figure 3 clearly shows that the regional PM_2.5_ level in Korea was greatly affected by the LRTT from China. |
| 6. For spatial panel model, the predetermined spatial weight matrix is important. The author shall elaborate this procedure more carefully. | We have described in detail the process for finding the optimal spatial panel data model. As mentioned in the main text, we compared the Akaike Information Criteria (AIC) for the spatial error model (SEM) and the spatial autoregressive model (SAR) by testing seven different spatial weight matrices for the SEM and SAR models: queen contiguity, three distance-based (2, 4, and 8 k-nearest-neighbors (KNN)) and three kernel functions (uniform, triangular, and gaussian). We finally selected the SEM models with the 8-KNN spatial weight matrix as the most appropriate models, as they have the lowest AIC. |
| 7. As a suggestion, the author might want to try a dynamic spatial panel model, see Li, K. (2017). Fixed-effects dynamic spatial panel data models and impulse response analysis. Journal of Econometrics, 198(1), 102-121. | Thank you for your suggestion. Extension of the spatial panel-data model is considered as a future research topic, and the following is mentioned in the conclusion: “extending the statistical model to the spatiotemporal panel data model can be a future research consideration in order to take spatial as well as temporal correlation into account.” |
