# Supplementary material for: Effects of transboundary PM2.5 transported from China on the regional PM2.5 concentrations in South Korea: A spatial panel-data analysis
Source: PLoS One. 2023 Apr 13;18(4):e0281988. doi: 10.1371/journal.pone.0281988 (PMC10101448; doi:10.1371/journal.pone.0281988)
Supplement: Attachment — Submitted filename: Edited_Comment_reply_V2_1205_22.docx [file pone.0281988.s005.docx]

Reviewer's Responses to Questions

**Reviewer #1:**

• In line 307, it would be better to clarify the main predictor variables “LRTT of PM2.5 from China”. For some N, at time t, is LRTT of PM2.5 from China defined as the average PM2.5 LRTT of China cities on HYSPLT back-trajectory five days before? I do not find a formal definition of this predictor variable, though the author discusses LRTT of PM2.5 from China on page 9.

- We clearly define LRTT on p. 5 as follows: “this study uses the average PM_2.5_ concentration observed at the passage time in Chinese cities in the pathways identified by the HYSPLIT backward trajectories within 1,000 km from the receptor cities as the LRTT-related variable in the following analysis, which is obtained every 6 h according to the arrival time at the receptor cities.”

• The author should explain more about Figure 2. One question is that the closest China city is 250km far away from the receptor Korean cities, by Figure 1. How can the author get the weekly average PM2.5 concentrations observed in Chinese cities in the pathways at a distance of 250km?

- We have added a detailed explanation for Figure 2 as follows: “Since we have information on the x-y coordinates of the Chinese cities in the pathways and the receptor cities, and on the PM_2.5_ concentrations observed in Chinese cities at the passage time, it is possible to calculate the levels of PM_2.5_ in Chinese cities by distance to the receptor cities in Korea during the study period.”

• In line 243, I guess that the author is discussing Figure 5 instead of Figure 4.

- A revision has been made accordingly.

• In line 167, I cannot see Figure 2 indicates that as the westerly wind approaches Korea, it passes through these large cities.

- We changed the sentence to “Figure 2 indicates that as the air trajectories pass through these large cities, the LRTT PM_2.5_ concentrations have increased.”

**Reviewer #3:**

1a. Little is said about the monitoring stations in South Korea. I guess they record PM at a selected point in space. These data do not therefore represent the average PM concentrations in an area of study. A discrete spatial model, such as the one considered by the authors, is well suited for spatial areal data. The data of this study are instead geo-statistical data, i.e. they represent pointwise observations of a continuous spatial random field. A geo-statistical model should be considered for the statistical analysis.

- We thank you for your insightful comments. Although air quality monitoring stations collect pointwise air pollution levels, they are known to record pollution levels representing an area covering approximately 2 km radius (approximately 12–15 km^2^). Therefore, we assume that air pollution levels recorded by 396 monitoring stations in Korea represent air quality in urbanized areas.

As an aside comment: I don’t understand how spatial weight matrices were computed. Spatial weight matrices are defined according to a specific neighborhood structure, which in turn requires a spatial tessellation. We don’t have any tessellation here because data are collected at single points in space… 4. The computations of Table 2 depend on a specific spatial neighborhood structure, a priori defined. Which neighborhood structure was considered? See also major issue 1a.

- We constructed a spatial weight matrix (SWM) using the coordinates of the monitoring stations. Spatial weight matrices are divided into contiguity-based and distance-based. Distance-based SWM has three different types: 1) distance band, 2) K-nearest neighbor (KNN), and 3) kernel. In this study, seven different SWMs were constructed and tested: queen contiguity, three different KNN (2, 4, and 8 KNN) and three kernel functions (uniform, triangular, and gaussian). We finally selected 8-KNN as the best SWM by comparing the Akaike Information Criteria (AIC).

1b. As figure 3 clearly shows, data are temporally correlated, but only spatial autocorrelation was taken into account. Ignoring an important source of autocorrelation may lead to severe bias in the p-value computations

- We thank you for your valuable comments. We revised our model by including two lag variables to address serial dependence. We added lag variables of NO2 and SO2, which are key determinants of the regional PM_2.5_ concentration as the secondary precursor. We added the following sentence on p. 13: “In addition, we added two lag variables of NO_2_ and SO_2_, which are key determinants of the regional PM_2.5_ concentration as the secondary precursor to take into account serial dependencies.”

2. Wind direction is a circular variable and requires special care. It seems that the circular support of wind direction has been ignored, as it was included in the model such as the other covariates. This is not correct. When the dependent variable (PM) is linear and a covariate (wind direction) is circular, the regression line is a line wrapped around a cylinder! I would suggest to transform wind direction to a qualitative variable, by clustering value into a finite number of classes. More rigorous approaches should account for the literature of linear-angular regression. As an aside comment: how was average wind direction computed? The equation of a circular mean is different of the standard mean computation of a linear variable.

- We thank you for your valuable suggestion. We replaced the wind direction variable with three qualitative variables representing westerly wind direction by adding the following sentence on p. 13: “For the wind direction, three westerly wind directional dummy variables are included: west-southwest (WSW: 1 if the azimuth wind direction is between 213 and 281 and 0 otherwise), 2) west-northwest (WNW: 282 and 326), and 3) north-northwest (NNW: 327 and 360).”

3. A single spatial autocorrelation parameter for a dataset like this is far from realistic. Spatial dependence tend to vary over time and across space! More generally, model checking is a bit overlooked. What about predictive capability of the model?

- Thank you for your comment. Like other spatial panel data models in the literature, this study also used a spatial panel data model under the assumption that association between the values in nearby locations would be similar over time.

4. Although the model relies on the normality assumption of the dependent variable, this assumption was not tested. Is the distribution of PM normal?

- We thank you for your valuable suggestion. We made a log transformation of the dependent variable (local PM_2.5_ level) to address the normality issue. Due to the log transformation, the calculation method for the average effects of LRTT from China in Table 4 was also modified.

5. To be more convincing, the model should include an estimation of pollution from other geographical areas such as North Korea and Japan (not just China...). I guess that data from North Korea are unavailable, and this could be a fatal issue.

- As you point out, PM_2.5_ concentrations in Korea can be affected not only by China, but also by North Korea and Japan. Looking at the wind direction during the study period, the effect from Japan would seem to be negligible. Conversely, North Korea may have an influence, but data is not available. As a result of the spatial panel model analysis, the North Korean wind direction variable (NNW) is neither statistically significant nor has positive values, so the influence from North Korea does not seem to be significant.

SPECIFIC ISSUES
1. Lines 167-168: “Figure 2 indicates that as the westerly wind approaches Korea, it passes through these large cities, increasing the LRTT PM2.5 concentrations”. Figure 2 does not include wind!

- We changed the sentence to “Figure 2 indicates that as the air trajectories pass through these large cities, the LRTT PM_2.5_ concentrations have increased.”

2. Figure 2: instead of overlapping two time series, correlation plots would be more informative.

- Figure 2 compares PM levels of Korea and LRTT from China, which we think demonstrates visually the relationship between the two variables.

3. Figure 4a is a bit misleading, because of the circular nature of wind direction.

- Figure 4a is a graph showing the average wind direction obtained from the backward trajectory. We believe that this graph helps readers to understand the azimuth of LRTT.
