# Supplementary material for: Effects of transboundary PM2.5 transported from China on the regional PM2.5 concentrations in South Korea: A spatial panel-data analysis
Source: PLoS One. 2023 Apr 13;18(4):e0281988. doi: 10.1371/journal.pone.0281988 (PMC10101448; doi:10.1371/journal.pone.0281988)
Supplement: Attachment — Submitted filename: referee report.pdf [file pone.0281988.s002.pdf]

Referee report of “Effects of transboundary PM2.5 transported from Chinese cities on the regional PM2.5 concentrations in South Korea: A spatial panel-data analysis” by Myung-Jin Jun

Summary:
The paper tried to adopt a spatial panel model to analyze the relationships between the PM2.5 concentrations among a set of Chinese cities and Korean cities. The author claimed significant transboundary PM2.5 transported from Chinese cities to South Korea. Overall, the quantitative analysis and the conclusions of this paper are hardly justifiable.

Comments:

1. For such spatial dynamic analysis, the modeler shall include all endogenous variables in the model. The author only considered several cities from China and South Korea. It might be the case that cities from Japan and North Korea also contribute to this transboundary dynamic. Simple omission of these possible endogenous variables may lead to severe bias.

2. The author has a wrong interpretation of the coefficient rho and lambda. The significance of these coefficients only suggest there exits spatial dynamic among these cities. It does not deliver the information of transboundary direction.

3. The Hausman test to distinguish between fixed effect and random effect is largely applicable to cross sectional setting. I don’t see how the author applied it to spatial panel setting.

4. It is confusing to read the summary statistics of Table 1, where variables appear in both rows and columns.

5. The conjecture of transboundary effects from China to Korea based on Figure 2 is far from serious.

6. For spatial panel model, the predetermined spatial weight matrix is important. The author shall elaborate this procedure more carefully.

7. As a suggestion, the author might want to try a dynamic spatial panel model, see Li, K. (2017). Fixed-effects dynamic spatial panel data models and impulse response analysis. Journal of Econometrics, 198(1), 102-121.
